# Qualitative and quantitative analysis of self-care regarding sensory issues among people with neurodevelopmental disorders

**DOI:** 10.3389/frcha.2023.1177075

**Published:** 2023-05-02

**Authors:** Makoto Wada, Katsuya Hayashi, Kai Seino, Naomi Ishii, Taemi Nawa, Kengo Nishimaki

**Affiliations:** ^1^ Developmental Disorders Section, Department of Rehabilitation for Brain Functions, Research Institute of National Rehabilitation Center for Persons with Disabilities, Tokorozawa, Japan; ^2^ Information and Support Center for Persons with Developmental Disorders, National Rehabilitation Center for Persons with Disabilities, Tokorozawa, Japan; ^3^ Psychological Experiment Section, Department of Social Rehabilitation, Research Institute of National Rehabilitation Center for Persons with Disabilities, Tokorozawa, Japan; ^4^ Hospital of National Rehabilitation Center for Persons with Disabilities, Tokorozawa, Japan; ^5^ Department of Pediatrics, Niigata National Hospital, National Hospital Organization, Kashiwazaki, Japan

**Keywords:** sensory issue, selfcare, developmental disorder, quality of life, autism spectrum disorder, hypersensitivity

## Abstract

**Introduction:**

Issues in sensory processing (hereafter, sensory issues) associated with neurodevelopmental disorders are known to be particularly prominent from 6 to 9 years of age and are a critical issue in school life. These issues affect each individual's quality of life. Some of the issues are known to be relieved by self-care while some are not.

**Methods:**

To clarify the sensory issues that cannot be managed by self-care, this study examined self-care for sensory issues among people with neurodevelopmental disorders using a web survey. The survey encompassed questions about neurodevelopmental disorders, the sensory issues individuals experience, and the kind of self-care they perform. In the qualitative analysis, each was categorized by the type of sensory modality; we further scrutinized the descriptions of self-care, which were collected simultaneously, and examined how each problem was addressed.

**Results:**

Self-care was categorized as “physically blocking,” “leaving from,” “relaxing,” “devising,” “help from others,” “taking medication,” “coping with body,” “others,” or “could not cope.” Based on these findings, we quantitatively compared the frequency of sensory issues that could and could not be managed by self-care. Consequently, significantly higher percentages of the participants stated that they experienced difficulties in managing problems about “body representations,” “contact with humans,” “selective listening,” and “force control.” In contrast, significantly more participants stated that they could manage problems related to “loud sound” and “dazzling”.

**Conclusion:**

In this study, qualitative analysis allowed us to categorize methods of self-care for sensory issues, and quantitative research allowed us to identify issues that were difficult to manage. While it was possible to manage strong light and sound using sunglasses, earplugs, and so on, problems related to the senses of proprioception, selective attention, and so on were clearly difficult to manage.

## 1. Introduction

People with neurodevelopmental disorders experience various issues in sensory processing (hereafter, sensory issues). Regarding autism spectrum disorder (ASD), excessive or restricted responses to sensory stimuli (hypersensitivity and hyposensitivity, respectively) are described in the Diagnostic and Statistical Manual of Mental Disorders, fifth edition (DSM-5), in addition to two major characteristics of atypicality in social communication and restricted and repetitive behaviors or interests (1). Such sensory issues are serious problems because some of them directly degenerate the quality of life (QoL) (2) of each individual. Sensory issues in neurodevelopmental disorders are known to be particularly prominent from 6 to 9 years of age (3, 4), and are critical issues in school life for children with neurodevelopmental disorders.

Among neurodevelopmental disorders, 60%–90% of people with ASD experience several sensory issues (5–11). A typical sensory issue in individuals with ASD is auditory hypersensitivity, which is an excessive response to sound, and its prevalence is estimated to be approximately 40% (12, 13). Furthermore, tactile hypersensitivity, which is an excessive response to tactile stimuli, is common in individuals with ASD (7, 14). While these hypersensitivities have been identified, studies have also reported enhanced perceptual acuity of these sensory modalities (14, 15), suggesting that some kind of atypicality in neuronal modulation is involved. To date, the neural mechanism of sensory issues is not clearly understood, although various possibilities have been suggested, involving both peripheral and central nervous systems (16–18).

In addition to ASD, attention-deficit/hyperactivity disorder (ADHD) and specific learning disorder (SLD) may entail sensory issues. For example, people with ADHD also have high scores for sensory sensitivity and sensation avoidance (19); however, this is partly attributable to a potential overlap with ASD. Recent imaging studies have also indicated shared changes in ASD in the white matter and their connections with hypersensitivity (20, 21). In contrast, higher visual processing scores were observed among children with ADHD than children with ASD and typically developing (TD) children, while oral processing scores (related to somatosensory function) were highest among children with ASD (22). These studies suggest that there are shared, but partly distinct, characteristics of sensory issues between people with ASD and ADHD. Some people with SLD suffer from low reading ability, parts of which show distorted vision and visual hypersensitivity (Irlen syndrome) (23–25). We previously conducted a qualitative and quantitative survey of people with neurodevelopmental disorders (ASD, ADHD, and SLD) to determine the types of sensory issues that exist (26) by using free writing fields and multiple-choice questions. The results showed that while many participants stated auditory problems as the most distressful sensory issues, a significantly higher percentage of people with ASD reported greater distress due to tactile problems compared with people with other neurodevelopmental disorders (ADHD, SLD). However, a greater percentage of those with SLD reported that visual problems were more distressful than other sensory problems. A qualitative analysis of each problem revealed that in addition to distress from strong stimuli, there was an element of confusion owing to multiple stimuli generated simultaneously. It was also found that they experienced various difficulties with other sensory modalities such as vestibular and proprioceptive sensation, temperature, and pain. Thus, although sensory issues associated with various neurodevelopmental disorders have been shown to have some commonalities, they also show wide diversity, making it important to address them individually.

For parts of these sensory issues, there are various well-known self-care methods. Regarding auditory hypersensitivity, the use of earplugs, ear muffs, or noise-canceling headphones can help significantly reduce noise (27, 28). Regarding visual hypersensitivity, as typified by dazzling, sunglasses can be used to reduce light, which is expected to reduce annoyance. Uniquely colored glasses for people with Irlen syndrome, which entails dyslexia-like symptoms and visual hypersensitivity, contributed to the improvement of the relevant symptoms (25, 29). However, there are several objections regarding the same (30, 31). Additionally, several good practices are empirically known to alleviate tactile hypersensitivity associated with clothing, such as prickling sensations including the mutilation of tags. However, self-care for a wide variety of sensory issues has not been fully covered. Moreover, not all forms of self-care are considered good practices. It is important to comprehensively examine self-care for sensory issues to ascertain the beneficial practices and identify problems that are currently difficult to address to further identify issues and develop support methods.

Therefore, we aimed to categorize self-care for each sensory issue using qualitative research, and analyze quantitatively which issues are difficult to manage by self-care. We asked participants to report the self-care methods for different sensory issues, addressing how they manage their respective sensory issues. Information on how each problem is managed can be used to promote effective self-care methods. Moreover, identifying problems that are difficult to manage will be useful for determining the direction for future developments in support methods. Therefore, according to the categories of sensory issues in the previous study, we scrutinized the descriptions of self-care, which were collected from the identical participants in the previous study simultaneously (26), and examined how each problem was addressed. Responses regarding self-care were categorized to determine how each sensory issue was addressed. This study aimed to identify sensory issues that could not be addressed using self-care by the participants, and clarify the issues that need to be addressed in future research.

## 2. Materials and methods

### 2.1. Questionnaire

The web survey was included in the questionnaire, parts of which were previously reported regarding which categories of sensory issues were included (26). In the questionnaire, participants diagnosed with (or suspected to have) neurodevelopmental disorders (ASD, ADHD, SLD, intellectual disability, and others), which are defined as “発達障害” (developmental disorders) in the Act on Support for Persons with Developmental Disorders in Japan, were targeted. The web-survey was originally written in Japanese [Supplementary document 1; identical to that in Wada et al. (26)]. First, the study purpose was explained. After confirming their consent to participate in the study by ticking a check box, participants were asked to respond to the questionnaire (in the case of minors, their guardians needed to agree on their behalf). The questionnaire encompassed multiple-choice questions and free-writing fields. First, multiple-choice questions about the respondent's gender, age, responder, current position regarding employment or educational status, and diagnosis were presented [Supplementary Table S1; identical to Table 1 in Wada et al. (26)]. The participants were then required to choose the modality with the most distressful sensory issues and concretely describe the issue that they experienced (free-writing field). If participants recognized the second and third most distressing issues, similar questions were presented for these issues. Regarding sensory issues, each sensory modality was categorized by scrutinizing its description in the free-writing field [Figure 3 and Table 3 in Wada et al. (26)]. Regarding each issue, there was a free-writing field (about self-coping, “How do you manage the sensory issues you mentioned when they occur? Please answer this question. If you have no coping strategies, please write ‘none’”) that was not analyzed in the previous study. This study analyzes the relationship between sensory issues and self-coping methods (hereafter, self-care).

### 2.2. Participants

The questionnaire was posted on the website of the Developmental Disorders Information and Support Center of the National Rehabilitation Center for Persons with Disabilities, Ministry of Health, Labour, and Welfare of Japan. We received 432 responses between August 2018 and January 2019. Of these, 415 responses containing descriptions of sensory issues were included in the analysis. This study was reviewed and approved by the Ethics Review Committee of the National Rehabilitation Center for Persons with Disabilities (29-175, 30-154, 31-109, 2021-136) and was conducted in accordance with the Declaration of Helsinki and “Medical Research Guidelines for Humans” of the Ministry of Health, Labour and Welfare of Japan. Characteristics of the participants were identical to the previous study [Supplementary Table S1, corresponding to Table 1 in Wada et al. (26)]. The diagnoses (including suspicion) of the participants are shown in Table 1.

**Table 1:** Diagnoses of the participants.

ASD	164
ADHD	55
SLD	15
ASD + ADHD	76
ASD + SLD	11
ADHD + SLD	9
ASD + ADHD + SLD	19
ASD suspicion	8
ADHD suspicion	2
SLD suspicion	2
ASD + ADHD suspicion	3
Others	51
Total	415

“ASD” includes autism, Asperger's syndrome, and pervasive developmental disorders. “Others” includes intellectual disability, developmental coordination disorder, and other developmental disorders (participants were not specified).

### 2.3. Analysis

We conducted mixed methods research with an exploratory sequential design to analyze descriptions of self-care for each sensory issue in individuals with neurodevelopmental disorders.

For the quantitative research, we extracted elements of descriptions of self-care, and labeled and categorized the descriptions by using protocols based on the K-J method (32, 33) as follows.

First, we extracted the statements that were the target of the present analysis from the dataset of the previous study (26). Because this study aims to determine how each sensory issue was addressed, all elements of the most, second most, and third most distressful sensory issues were aggregated (1,376 descriptions in total). After excluding descriptions that could not be classified or were less than 15 in each category of the sensory issues, 1,170 descriptions of sensory issues were included in the analysis.

The free-writing field (“How do you manage the sensory issues you mentioned when they occur? Please answer this question. If you have no coping strategies, please write ‘none’”) was analyzed afresh using a similar method as our previous study for categorizing sensory issues themselves (26). Regarding the 1,170 descriptions, four authors (two parents of children: T. N and N. I; two researchers: M. W and K. S for medical science and welfare, respectively) extracted and labeled the descriptions of self-care. When a description contained more than two elements (e.g., “Sometimes, in noisy rooms, I use earplugs to reduce sound, and occasionally step away for a break.”) they were aggregated in each category. The labeled descriptions were divided into several categories according to type of coping. Discrepancies in categorization among the four individuals were discussed until they reached a satisfactory decision.

Based on the categories of self-care, we conducted the following quantitative analysis. Tables 2, 3 present typical descriptions in each category and the frequency of categorized self-care for each sensory issue respectively. To identify issues that were difficult to address, the frequency of the sensory issues that could be managed and those that could not were compared. *Χ*^2^ tests (test of independence) were further conducted to test statistical differences between the categories of sensory issues. Data analysis was conducted using Microsoft Excel (Office2019, Microsoft, Redmond, WA, USA).

**Table 2:** Examples of self-care for sensory issues.

Physically blocking	サングラスをする ノイズキャンセリングのイヤフォンをつける タグを切る マスクをしたり、鼻や口をハンカチでおさえたり Wearing sunglasses. Wearing noise-canceling earphones. Cutting off tags of clothes. Wearing a mask or covering the nose and mouth with a handkerchief.
Devising	白い紙の上にルーラーを乗せて読む その音を聞くことにだけ集中する。音が止んだのがわかるまで気を抜かない Reading with a ruler on a white paper. Concentrating only on hearing the sound.
Leaving the area	その場からなるべく離れたり避けたりする 距離をとる。少し間を開ける 食べない 食べられるもののみにする Moving away from the area as much as possible. Keeping a distance. Only eating what I can eat.
Relaxing	休めるときはやすむ Resting when I can.
Taking medication	パキシル錠を服用。 薬効が出るまでは目を瞑って耐える ストラテラを服用して何とか話を理解できるように努めている 保湿剤などの水分やクリームを塗る Taking paroxetine tablets. I meditate and endure until the medication takes effect. Taking atomoxetine in an effort to relatively understand what is being said. Applying moisturizers.
Help from others	周りに環境調整してもらう キーパーソンと過ごす時間を設け、気分を落ち着かせる Asking the person near me to adjust the environment. Setting time aside to spend with key persons to calm them down.
Coping with body	見えている世界をなんとか自分の知っている世界とすりあわせる 実際に体験させる Relatively reconcile the world I see with the world I know. Let them actually experience it.
Others	周りの人の声が気になって耐えきれない時は、口頭で注意してしまうことがある When I cannot bear to hear the voices of others around me, I sometimes verbally warn them.
Could not cope	とにかく我慢する どうしようもない I'll put up with it anyway. I cannot help it.

Examples of descriptions of self-care for sensory issues are shown as original sentences in Japanese accompanied by English translations.

**Table 3:** Aggregate results of categories of self-care for each sensory issue.

		Physically blocking	Leaving from	Relaxing	Devising	Help from others	Taking medication	Coping with body	Others	Could not cope	Sum
Visual	Dazzling	62	12	7	7	3	4	2		9	106
	Confusion of visual information	11	5	5	3	3		1		4	32
	Specific visual stimulus	10	5		4	1				5	25
	Difficulty in reading	1	2	1	11	3				5	23
	Abnormal vision	3	2	2	2	4	4			3	20
Auditory	Specific sounds	93	57	19	15	9	5	6	2	37	243
	Selective listening	36	19	10	19	12	2	3		33	134
	Many sounds	32	23	9	3	2	3	2		18	92
	Loud sounds	27	16	9		4	1	2		1	60
	Sudden sounds	14	6	3	1	3		1		2	30
Tactile	Clothes	32	8		1		2			3	46
	Contact with humans	6	3	2	1	2	1	1		9	25
	Specific target	16	5	1	3		1	1		7	34
Olfactory	Specific odors	41	49	5	4	3	2			22	126
Proprioception	Body representations				3			4		13	20
	Force controls			3	3	3		4		7	20
Vestibular	Dizziness		2	10	1		1			5	19
Others	Temperature	2	5	13	12	4	9	1		8	54
	Air pressure	3	6	17	3	3	7			6	45
	Multisensory	4	4	3			1			4	16

The rightmost column of the table shows the number of occurrences for each category of sensory issues.

## 3. Results

### 3.1. Free writing about self-care for each sensory issue

We scrutinized the descriptions of self-care and examined how each sensory issue (1,170 descriptions) was addressed. The self-care methods for each sensory issue could be categorized as: (1) Physically blocking distressful sensory stimuli by using earplugs, sunglasses, or other means (“physically blocking”); (2) Leaving from places where there are distressful sensory stimuli (“leaving from”); (3) Taking a temporary break or relaxing (“relaxing”); (4) Devising some way to make the situation better (“devising”); (5) Getting help from others (“help from others”); (6) Using medication to alleviate the vexation (“taking medication”); (7) Getting used to the situation or being able to handle it well, on the body side (“coping with body”); (8) Other means (“others”); and (9) There is no appropriate way to cope with the situation (“could not cope”). Table 2 shows typical descriptions of each category.

**Figure 1:**
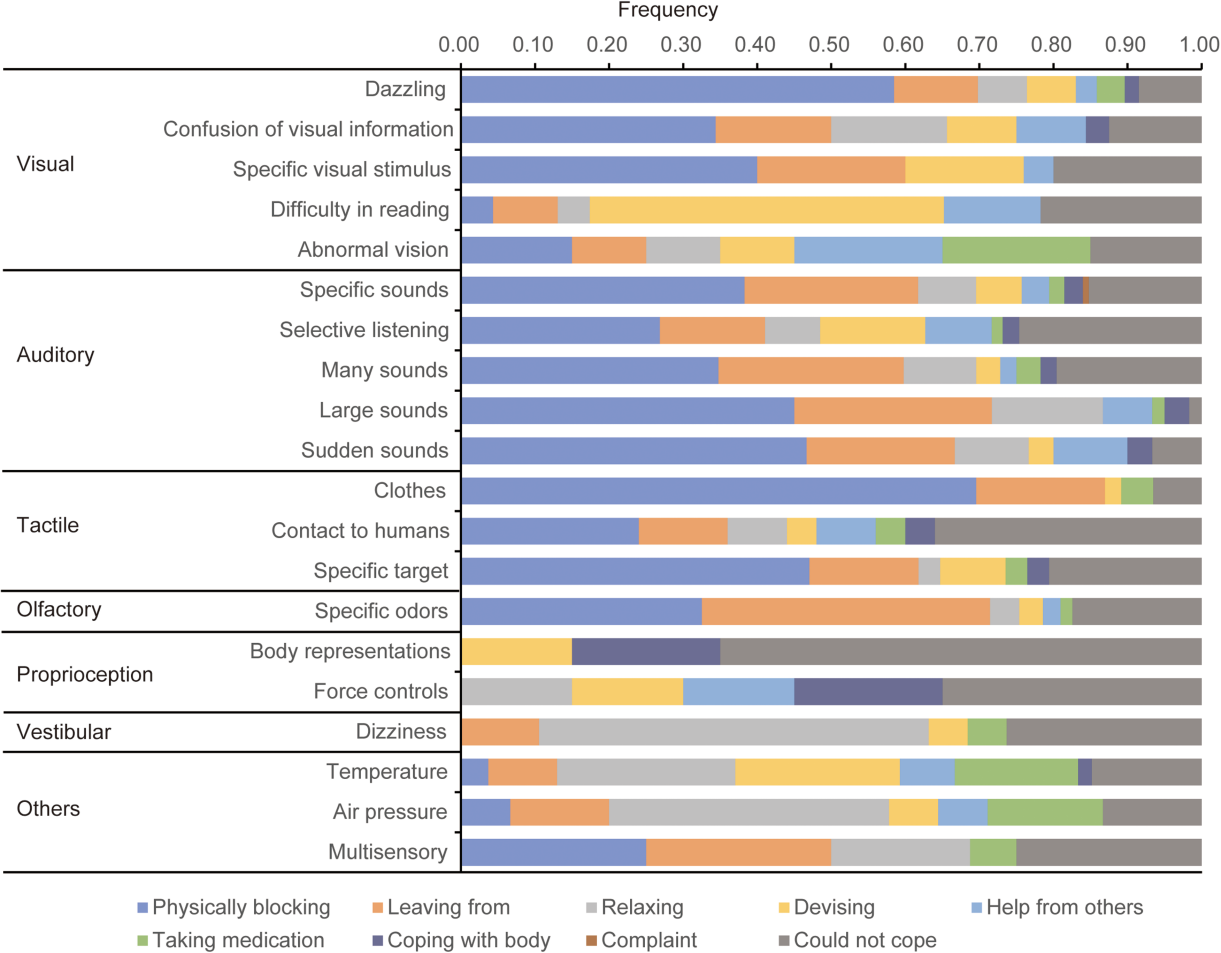
Frequency of categories of self-care for each sensory issue.

Figure 1 and Table 3 show the frequency/aggregate results of occurrence of the categorized self-care methods for the categories of sensory issues [Corresponding to the category of Table 3 in Wada et al. (26)] that contained 15 or more responses, respectively. These results suggested that the blocking is effective for strong or noxious stimuli.

### 3.2. Identifying sensory issues that were difficult to address

To identify issues that were difficult to address, the frequency of the sensory issues that could be managed and those that could not were compared. *Χ*^2^ tests (test of independence) were further conducted to examine statistical differences between the categories of sensory issues; the *Χ*^2^ test showed significant differences (*Χ*^2^ = 74.7, *df* = 19, *p* < 0.00001). Residual analysis (Table 4) showed that a significantly higher percentage of participants reported difficulty in managing problems related to body representations (“body representations,” adjusted residual = 5.72, *p* ≤ 0.0001), fear of physical contact with humans (“contact with humans,” adjusted residual = 2.52, *p* = 0.012), difficulty in the selective listening (“selective listening,” adjusted residual = 2.43, *p* = 0.015), and impairment in force control (“force control,” adjusted residual = 2.13, *p* = 0.033). However, significantly more participants answered that they could manage problems related to dislike toward loud sounds (“loud sound,” adjusted residual = −3.27, *p* = 0.0011) and the dislike of dazzling (“dazzling”, adjusted residual = −2.49, *p* = 0.013).

**Table 4:** Results of the residual analysis comparing the appearance rates that could not be managed among each sensory issue.

		Could cope		Could not cope	
		Residual	*p*	Residual	*p*
Visual	Dazzling	2.49	0.013	−2.49	0.013
	Confusion of visual information	0.71	0.48	−0.71	0.48
	Specific visual stimulus	−0.38	0.71	0.38	0.71
	Difficulty in reading	−0.59	0.56	0.59	0.56
	Abnormal vision	0.26	0.79	−0.26	0.79
Auditory	Specific sounds	0.91	0.36	−0.91	0.36
	Selective listening	−2.43	0.015	2.43	0.015
	Many sounds	−0.63	0.53	0.63	0.53
	Large sounds	3.27	0.0011	−3.27	0.0011
	Sudden sounds	1.55	0.12	−1.55	0.12
Tactile	Clothes	1.96	0.051	−1.96	0.051
	Contact to humans	−2.52	0.012	2.52	0.012
	Specific target	−0.53	0.59	0.53	0.59
Olfactory	Specific odors	−0.09	0.93	0.09	0.93
Proprioception	Body representations	−5.72	<0.0001	5.72	<0.0001
	Force controls	−2.13	0.033	2.13	0.033
Vestibular	Dizziness	−1.06	0.29	1.06	0.29
Others	Temperature	0.47	0.64	−0.47	0.64
	Air pressure	0.70	0.49	−0.70	0.49
	Multisensory	−0.84	0.40	0.84	0.40

Residual analysis was conducted because the *X*^2^ test (test of independence) revealed significant differences between the categories of sensory issues.

## 4. Discussion

This study investigated the categories of self-care for various sensory issues among people with neurodevelopmental disorders using a web-survey, and examined (1) categories of self-care for the sensory issues (Qualitative analysis, Table 2), (2) the frequency of each self-care for each sensory issue (Quantitative analysis, Figure 1 and Table 3), and (3) the frequency of the sensory issues that could be managed (Quantitative analysis, Table 4).

### 4.1. Categories of self-care for the sensory issues and frequencies

As described in the Results section, several categories of self-care for sensory problems were identified. Of these, the responses of “physically blocking,” and “leaving from” may attenuate the unpleasant stimulus. If a participant felt uncomfortable in a situation with a sensory stimulus, the common form of self-care was to block or move away from the stimulus, for example, by using noise-canceling headphones, sunglasses and so on. This result supports the benefits of noise-canceling headphones, as shown in previous studies (27, 28). Whereas, “relaxing,” and “taking medication” may attenuate discomfort by reducing anxiety. Both are countermeasures against sensory sensitivity. With regard to medication, it should be carefully considered whether medications for ADHD symptoms are effective in alleviating sensory sensitivity.

In contrast, among problems other than uncomfortable stimuli, such as difficulty in reading or listening, and motor control problems involving proprioception, we observed various attempts at self-care, including “devising,” “help from others,” and “coping with body.” For problems involving the internal state of the body, such as those related to vestibular sensation (“dizziness”), temperature, and pressure sensation, the first emphasis seemed to be on relaxation.

### 4.2. Sensory issues that could be managed

In the quantitative analysis, we further examined the percentage of individuals who reported that they could or could not manage each sensory issue. As a result, significantly more respondents answered that they could relatively manage problems related to “Loud sound,” and “Dazzling” (mainly by “Physically blocking”). Regarding physically strong stimuli, a physical blockade may be effective and relatively easy to manage against hypersensitivity toward strong sensory stimuli. It should be noted, however, that when multiple sensory sensitivities coexist, these self-care measures may not be well utilized. As we have reported previously, multiple sensory problems often coexist, such as auditory and tactile hypersensitivity (26). Thus, there may be a situation in which the means for blocking out noise lights, such as noise-canceling headphones or sunglasses, may be difficult to use because of tactile sensitivity. Therefore, it is important to carefully examine the methods used in these situations as well.

### 4.3. Sensory issues that could not be managed

By identifying the sensory issues which could not be managed with self-care, it is possible to identify issues that need to be addressed using new support methods in future. Among these, significantly higher percentages of participants reported difficulty in managing problems about “body representations,” “contact with humans,” “selective listening,” and “force control.” Problems related to proprioception and difficulty in selective listening are not solved by simply eliminating noisy stimuli; therefore, difficulties in managing these problems have arisen. Development of appropriate support methods in future is important. In other words, it will be useful to develop support and rehabilitation methods that strengthen the perception of specific sensory stimuli and promote awareness, rather than diminishing the stimuli as a whole.

Increasing awareness of specific sensory stimuli is considered important not only for issues about proprioception, but also for issues about selective listening. However, regarding the problem of selective listening, there were relatively many responses about using means of physically blocking, such as noise-canceling headphones. Accordingly, this can be solved by eliminating background noise. However, such noise-canceling functions are designed to eliminate non-human noise and cannot reduce the difficulties caused by multiple people speaking simultaneously. Therefore, it is necessary to develop support methods to address this problem. Recently innovated smart earplugs, smartphones, and other smart devices have functions that emphasize the voice of the person in front of the user or the voice of the dialogue partner. Using these devices to address the problem of selective listening may provide effective assistance.

However, it is difficult to reduce problems related to physical contact with other people by self-care, because of the presence of a partner. It is considered important to promote understanding and awareness among the surrounding people by enlightening them that there are some types of hypersensitivity that do not favor skin contact. It is possible that after the completion of this survey, there has been a reduction in this problem owing to the increased understanding of avoiding physical contact, such as handshakes, owing to the prevalence of new lifestyle habits associated with the COVID-19 epidemic.

As described above, the present survey quantitatively demonstrated sensory issues that are difficult to alleviate through self-care at present, thereby identifying targets for the development of new support and rehabilitation methods and for the promotion of awareness.

### 4.4. Limitations of the present study

As previously described, this study has some limitations (26). First, participants' diagnoses were based on self-report, which included a small number of cases of “suspected at the clinic.” While this is not likely to be a serious problem when considering self-care for each of the sensory issues, it is a potential limitation. Second, the survey was limited to individuals who were aware of their sensory issues and participated voluntarily. Therefore, sample bias may exist when compared with the average population with neurodevelopmental disorders. In addition to the possibility of differences in the manifestation of sensory issues, this may reflect differences in interest in the topic and hesitancy to participate in a research study. Third, the participants' cognitive capacity was not controlled because they were broadly recruited from a website. Thus, surveys with diverse samples should control for variables such as gender, social background, and cognitive capacity to reduce sample bias in future.

## 5. Conclusion

In this study, significantly higher percentages of respondents reported difficulty in managing the problems about “body representations,” “contact with humans,” “selective listening,” and “force control.” In contrast, significantly more participants answered that they could manage problems related to “Loud sound” and “Dazzling.” While it was possible to manage strong light and sound using sunglasses, earplugs, and so on, it was clear that problems related to the senses of proprioception and selective attention were difficult to manage and avoid in the case of interpersonal contact.

## Data Availability

The raw data supporting the conclusions of this article will be made available by the authors, without undue reservation.
