# Supplementary material for: Qualitative and quantitative analysis of self-care regarding sensory issues among people with neurodevelopmental disorders
Source: Front Child Adolesc Psychiatry. 2023 May 2;2:1177075. doi: 10.3389/frcha.2023.1177075 (PMC11731913; doi:10.3389/frcha.2023.1177075)
Supplement: Supplementary file 1 [file Datasheet1.docx]

**Supplementary tables and documents**

**Supplementary Table 1.** Participants’ demographics. Following tables were identical to table 1 in Wada et al. 2023).

|  | A. Gender | |  |
| --- | --- | --- | --- |
|  |  |  |  |
|  |  |  |  |
|  | **Male** | 172 |  |
|  | **Female** | 230 |  |
|  | **Not specified** | 13 |  |
|  | **Total** | **415** |  |
|  |  |  |  |

|  | B. Age range (years) | |  |
| --- | --- | --- | --- |
|  |  |  |  |
|  |  |  |  |
|  | **< 6** | 7 |  |
|  | **< 12** | 50 |  |
|  | **< 18** | 56 |  |
|  | **< 23** | 38 |  |
|  | **< 30** | 65 |  |
|  | **< 40** | 61 |  |
|  | **< 50** | 84 |  |
|  | **< 60** | 51 |  |
|  | **< 70** | 3 |  |
|  | **Total** | **415** |  |
|  |  |  |  |

|  | C. Responder | |  |
| --- | --- | --- | --- |
|  |  |  |  |
|  |  |  |  |
|  | **In person** | 274 |  |
|  | **Parent** | 114 |  |
|  | **Supporter** | 27 |  |
|  | **Total** | **415** |  |
|  |  |  |  |

| D. Current position |  |  |
| --- | --- | --- |
|  |  |  |
|  |  |  |
| **Preschool** | 12 |  |
| **Elementary school** | 53 |  |
| **Junior high/high school** | 46 |  |
| **Special support schools** | 14 |  |
| **Higher education** | 25 |  |
| **Working** | 174 |  |
| **Working as a welfare worker** | 40 |  |
| **Other** | 79 |  |
| **Total (including duplicated responses)** | 443 |  |
|  |  |  |
|  |  |  |

*Note.* For current position, 28 duplicated responses (14 participants) are included.

**Supplementary document 1.** Questionnaire for the present study.

質問1：

本研究に参加することに同意して頂けますか。「はい」を選択した上で、「回答する」ボタンを押した段階で同意いただいたことになります（完全に匿名データですので、対象が特定できないため送信後の削除は困難です）。

　研究に関する説明や連絡先については、研究説明書（リンク先）をご覧ください。

はい

いいえ

Question 1.

Do you agree to participate in this study? If you select "Yes" and click the "Answer" button, you have given your consent (since the data is completely anonymous, it is difficult to delete it after transmission because the subject cannot be identified).

　For more information about the research and contact information, please refer to the research instructions (link).

YES

NO

質問2：

ご回答されているのは発達障害のある方ご本人ですか。それとも発達障害のある方の家族・支援者ですか。該当する項目を選んでください。

＊発達障害のある方の支援者である場合、具体的に１人の当事者の方についてご回答ください。

＊代理で回答されている場合、対象となる発達障害のある当事者ご本人（以下「当事者」）お一人を念頭にご回答ください。

当事者本人

家族（親・兄弟姉妹等）

支援者

Question 2.

Are you answering this question as a person with a developmental disability? Or are you a family member or supporter of a person with developmental disabilities? Please select the appropriate item.

If you are a supporter of a person with developmental disabilities, please answer for one person in particular.

If you are answering on behalf of someone with a developmental disability, please answer with one person in mind (the "person").

In person

Parent

Supporter

質問3：

あなた（代理で回答されている場合、発達障害のある当事者ご本人：以下「当事者」）の年齢を教えてください。

６歳未満

６～１１歳

１２～１７歳

１８～２２歳

２３～２９歳

３０～３９歳

４０～４９歳

５０～５９歳

６０～６９歳

７０歳以上

Question 3.

What is the age of you?

Under 6 years old

6-11 years old

12-17 years old

18-22 years old

23-29 years old

30-39 years old

40-49 years old

50-59 years old

60-69 years old

Over 70 years old

質問4：

あなた（当事者）の主なご所属は、以下のどちらに当たるか教えてください（複数回答可）。

就学前（含幼稚園・保育所等）

小学校

中学・高校

特別支援学校

高等教育機関（大学・大学院・短大・専門学校等）

仕事をしている

福祉的就労をしている

それ以外

Question 4,

Please tell us which of the following is your main affiliation (multiple answers are acceptable).

Preschool (including kindergarten, nursery school, etc.)

Elementary school

Junior high school and high school

Special support schools

Higher education institutions (universities, graduate schools, junior colleges, vocational schools, etc.)

Working

Working as a welfare worker

Other

質問5：

あなた（当事者）の性別を教えてください。

男性 女性 その他・回答しない

Question 5.

What is your gender?

Male

Female

Not specified

質問6：

あなた（当事者）は発達障害などの診断を受けましたか（公的機関による判定を含む）？該当する項目を選んでください（複数回答可）。

自閉スペクトラム症（自閉症・アスペルガー症候群・広汎性発達障害を含む）

注意欠陥多動性障害

学習障害

知的障害

その他の発達障害

不明（未診断・疑いを含む）

Question 6.

Have you received a diagnosis of developmental disorders (including a judgment by an official institution)? Please select all that apply (multiple answers are acceptable).

Autism spectrum disorder (including autism, Asperger syndrome, and pervasive developmental disorder)

Attention-deficit/hyperactivity disorder (ADHD)

Learning disorder

Intellectual disorder

Other developmental disabilities

Unknown (including undiagnosed or suspected)

質問7：

質問6で「その他の発達障害」「不明（未診断・疑いを含む）」を選択した方は、具体的な診断名（公的機関による判定を含む）をお答えください。

Question 7.

If you selected "other developmental disabilities" or "unknown (including undiagnosed or suspected)" in question 6, please provide the specific name of your diagnosis (including judgments by official institutions).

質問8：

あなた（当事者）が、今、最もつらいと感じている感覚の問題は次のうちどの感覚にあたりますか？

＊わからない場合は、「その他」にチェックを入れて、質問９に具体的にお書きください。

視覚（見え方の問題：ちかちかした光が苦痛など）

聴覚（聞こえ方の問題：特定の音が苦手など）

触覚（触ったときの問題：服のタグが苦痛など）

味覚（味の問題：極端な偏食があるなど）

嗅覚（においの問題：芳香剤が苦痛など）

固有覚（身体の問題：力加減がわからないなど）

前庭覚（平衡感覚の問題：めまい・ふらつきなど）

その他（温度など）・特定不能（気象条件など）

Question 8. <Results of a survey on this question was already published>

Which of the sensory issues do you feel most painful now?

If you don't know, please check "Other" and specify in question 9.

Visual (visual problems, e.g., distress from flashing lights)

Auditory (auditory problems, e.g., discomfort with certain sounds)

Tactile (tactile problems, e.g. tags on clothes are painful)

Taste (taste problem, e.g., extreme picky eaters)

Odor (odor problems: air fresheners are painful, etc.)

Proprioception (proprioception problems, e.g., not knowing how to control force)

Vestibular (vestibular problems: dizziness, lightheadedness, etc.)

Other (e.g., temperature) or unspecified (e.g., weather conditions)

質問9：

質問8で「その他（温度など）・特定不能（気象条件など）」を選択した方は、具体的な感覚（その他の場合）や状況（特定不能の場合）をお答えください。

Question 9. <Results of a survey on this question was already published>

If you selected "other (e.g. temperature) or unspecified (e.g. weather conditions)" in question 8, please describe the specific sensation (if other) or situation (if unspecified).

質問10：

ご回答いただいた感覚の問題とは、具体的にどのようなものですか？また、それが起こるきっかけや理由は何ですか（例：「ありとあらゆる音が聞こえて頭が混乱する」など）。

Question 10. <Results of a survey on this question was already published>

Please describe concrete examples of your sensory issues. What are the triggers and reasons why they occur (e.g., "I hear all kinds of sounds and my mind gets confused")?

質問11：

ご回答いただいた感覚の問題が起こるのは、どんなときですか？（複数回答可）

起床後 通学／通勤中 授業中 仕事中 雑談中 運動中 食事中 就寝中 その他

Question 11. <This question is preliminary investigation for future studies>

When do the sensory issues you described occur? (Multiple answers allowed)

After waking up

Commuting to school/work

In class

At work

During chatting

During exercise

While eating

While sleeping

Other

質問12：

質問11で「その他」を選択した方は、具体的な場面をお答えください。

Question 12. <This question is preliminary investigation for future studies>

If you selected "other" in question 11, please describe specific situations.

質問13：

ご回答いただいた感覚の問題が起こるのは、どんな場所ですか？（複数回答可）

自宅 レストラン（飲食店） お店（飲食店を除く） 学校 職場 イベント 路上 公共交通機関 自動車（公共交通機関を除く） その他

Question 13. <This question is preliminary investigation for future studies>

What are the places where the sensory issues you described occur? (Multiple answers allowed)

Home

Restaurants (eateries)

Stores (excluding restaurants)

School

Workplace

Event

On the street

Public transportation

Automobile (excluding public transportation)

Other

質問14：

質問13で「その他」を選択した方は、具体的な場所をお答えください。

Question 14. <This question is preliminary investigation for future studies>

If you selected "other" in question 13, please give the specific location.

質問15：

ご回答いただいた感覚の問題のつらさを「０（つらくない）～５（耐え難い）」で表現すると、どれにあたりますか？一番つらいときを想定して０～５を選択してください。

０（つらくない） １ ２ ３ ４ ５（耐え難い） 回答不能

Question 15. <Results of a survey on this question was already published>

On a scale of 0 (not painful) to 5 (unbearable), which of the following describes the severity of the sensory issues you indicated? Please select 0-5 for the most difficult time.

0 (not painful)

1

2

3

4

5 (unbearable)

Unable to answer

質問16：

ご回答いただいた感覚の問題によって生じる行動の制限を「０（行動は制限されない）～５（何も行動できない）」で表現すると、どれにあたりますか？一番つらいときを想定して０～５を選択してください。

０（つらくない） １ ２ ３ ４ ５（耐え難く、何も行動できない） 回答不能

Question 16. <Results of a survey on this question was already published>

On a scale of 0 (no activity is restricted) to 5 (no activity is possible), which of the following would you classify as a limitation of action caused by the sensory issues you indicated? Please select 0-5 for the most difficult time.

0 (not hard)

1

2

3

4

5 (unbearable, unable to take any behavior)

Unable to answer

質問17

ご回答いただいた感覚の問題が起こったとき、どのように対処していますか？具体的にお答えください。対処法がない場合は、「なし」と書いてください。（例：「イヤホンを付けて目を閉じて、気分を落ちつかせる」など）

Question 17.

How do you manage the sensory issues you mentioned when they occur? Please answer this question. If you have no coping strategies, please write’ none.’ (e.g., "Put on earphones and close your eyes to help you feel calm.")

質問18：

その他ご意見、ご感想がありましたら、ご記入ください。

＊こういうものがあれば、あるいは、こういう対応をしてもらえれば、という要望や提案などがあればお書きください。

Question 18. <This question is for the feedback on the survey>

Please provide any other comments or suggestions you may have.

*Please write any requests or suggestions you have for this kind of thing, or for this kind of response.

「２番目、３番目につらいと感じていることに回答しない方は、回答しなくて構いませんので質問19以降は飛ばして一番下の『回答する』ボタンをクリックしてください。」

If you do not want to answer the second or third hardest question, you do not have to answer it, so please skip question 19 and onward and click the "Submit" button at the bottom.

<Questions 8 through 17 were repeated for the second and third most painful sensory issues.>
